# Climate-Informed Patient Care as a Social Determinant of Health

**DOI:** 10.1001/jamahealthforum.2024.0095

**Published:** 2024-01-05

**Authors:** Anna Goldman, Benjamin D. Sommers

**Affiliations:** Section of General Internal Medicine, Department of Medicine, Boston Medical Center, Boston, Massachusetts; Department of Health Policy and Management, Harvard T. H. Chan School of Public Health, Harvard University, Boston, Massachusetts

The summer of 2023 was marked by devastating climate events: rapidly proliferating wildfires in Lāhainā, Maui, destructive floods across the northeastern US, and extensive forest fires in eastern Canada that filled the air across North America with suffocating smoke. Communities across the US contended with extreme heat, particularly in July 2023, when global temperatures were the hottest ever recorded. The current science is unequivocal: climate change poses an existential danger to human health and to the planet’s ecosystems. More than 250 health care journals across the world have cited climate change as the single greatest threat to public health.^1^

Climate change has an outsized effect on the health of children, older adults, and people who primarily work outdoors—in the form of heat stress, physical injury sustained during extreme weather, and increases in water and vector-borne infectious diseases.^2^ Poverty and structural racism intersect with climate change to compound risks for racial and ethnic minority groups and low-income communities. For example, these communities experience a higher burden of air pollution caused by fossil fuel combustion, which is strongly linked to respiratory disease, cardiovascular disease, and dementia.^3^

Across the US health care system, social determinants of health (SDOH) are increasingly recognized as key drivers of health outcomes for historically marginalized populations.^4^ Federal programs incentivize health care organizations and clinicians to screen for health-related social needs, such as food insecurity, housing instability, and unemployment—and to address these needs by connecting clients to social services programs. Although debate exists as to whether the health care sector is optimally suited to address SDOH, such efforts have nevertheless taken on a growing role, with more than $2.5 billion invested in screening and programs to address SDOH from 2017–2019.^5^

Climate change intensifies SDOH challenges in nearly every domain. For example, because of the historical practice of redlining (in which the availability of affordable mortgages were intentionally limited in neighborhoods with predominantly Black residents), Black individuals in the US are more likely to live in high-risk flood zones, making them more vulnerable to displacement as sea levels rise. Climate-related increases in food prices, which are driven by volatile temperatures, prolonged droughts, and heat waves leading to crop failures, hit lower-income communities hardest, where food insecurity is already rising.^6^

The recognition of climate change as a social determinant of health has at least 3 implications. First, this recognition creates a mandate for the health care system itself to rapidly decarbonize. Health care activities contribute 8.5% of US greenhouse gas emissions.^7^ It makes little sense for the health care system to invest billions in mitigating SDOH, while at the same time contributing to a deepening climate crisis through its own carbon-generating operations. During the process of decarbonizing, the health care sector must also increase the resilience of health care infrastructure so that the system can continue to operate smoothly during extreme weather events and other periods of instability.

Second, health care providers must screen for and address SDOH that are directly related to climate change (eg, access to air conditioning during heat waves can save lives, particularly for frail older adults and those with debilitating chronic conditions). Third, health systems seeking to address SDOH should view program development as an opportunity to meet the dual aims of improving SDOH and furthering progress toward climate goals.

Programs that address SDOH can help advance environmental justice via many avenues. For example, health systems can work with food banks to help strengthen local food systems and shift offerings toward healthier and more sustainable food options such as plant-based proteins instead of animal-derived products. Health systems engaged in creating affordable housing can prioritize low emissions or net-zero building approaches. Programs to connect patients to job training and employment can prioritize opportunities in the renewable energy sector.

Earlier this year, Boston Medical Center (BMC) announced a new initiative, the Clean Power Prescription program, that provides an example of balancing a dual mission of addressing SDOH in tandem with environmental justice. The program uses solar power generated at BMC to distribute solar credits to patients who report difficulty paying their utility bills. Like many other safety net hospitals, BMC treats a large share of patients covered by public insurance (a population with a high burden of health-related social needs). Since 2017, BMC has screened patients for housing instability, food insecurity, and inability to afford energy bills. Energy insecurity (when people struggle to cover the cost of home energy use) is considered an SDOH because energy is required to maintain a safe and comfortable household temperature, to operate medical equipment such as nebulizer machines, to safely store medications that require refrigeration, and to store and prepare fresh and nutritious food.^8^

The Clean Power Prescription program offers an opportunity for patients experiencing energy insecurity to receive assistance with energy bills while also benefiting from a renewable energy source. Through the program, a solar array on the roof of a hospital building generates virtual net metering solar credits, which will then be applied directly to patients’ electric bills. Virtual net metering is a mechanism in which energy output from a renewable energy system is measured and valued by a utility company. The utility company credits the owner of the renewable energy infrastructure, who can then direct the utility to allocate these credits to other account holders, which is how BMC will provide solar energy credits to its patients. In the pilot phase, 80 to 100 households enrolled in BMC’s Medicaid managed care plan will receive a $50 credit monthly for 12 months. Patients who participate in the Clean Power Prescription program will be offered home energy efficiency consultations to reduce the risk of energy insecurity after leaving the program.

This innovative approach was made possible by the Inflation Reduction Act’s “Low-Income Communities Bonus Credit program,” a tax credit incentivizing solar projects that direct clean energy to low-income households. In the coming months, BMC aims to scale up this effort by inviting other businesses and property owners to partner with the program by contributing energy credits from solar arrays they own or plan to build. By contributing at least 50% of the credits generated by a solar array, partner organizations would become eligible to receive the Low-Income Communities Bonus Credit, which can offset the cost of solar array installation by up to 70%. Efforts such as these offer a pathway (with adequate investment of resources) to improve patient well-being and outcomes, while also working toward environmental justice.

Climate change is perhaps the defining societal challenge of the coming decades. At the same time, dismantling centuries of health disparities within the US is one of the defining health care challenges of our time. The ever-increasing influence of climate change on SDOH and health disparities requires integrated policy approaches to both problems. The increasingly frequent disruptions caused by climate change may overwhelm the efforts of the health care system to deal with SDOH if those latter efforts ignore the changing environment. The pathway to a more just health care system requires climate justice at the same time.
